# Simple Technique to Address Graft-Tunnel Mismatch in Bone−Patellar Tendon−Bone Anterior Cruciate Ligament Reconstruction

**DOI:** 10.1016/j.eats.2025.103546

**Published:** 2025-04-25

**Authors:** Nathan Moroski, Geoff Marston, Joshua R. Eskew

**Affiliations:** Department of Orthopaedic Surgery, Prisma Health Upstate, Seneca, South Carolina, U.S.A.

## Abstract

Graft-tunnel mismatch during anterior cruciate ligament reconstruction using bone−patellar tendon−bone autograft is a significant problem, and it is imperative that the orthopaedic surgeon knows how to address this intraoperatively. Graft-tunnel mismatch has been reported to be as high as 20% during anterior cruciate ligament reconstruction with bone−patellar tendon−bone autograft. We present a technique that is easily reproducible using a simple whipstitch for backup fixation after trimming the tibial-sided bone block that gives reliable tension on the graft without necessitating adjustment of femoral fixation.

Anterior cruciate ligament (ACL) rupture is a very common injury in active and athletic patients, and ACL reconstruction is the preferred surgical option for the ACL-deficient knee with functional instability.^1^ Although there are multiple different options for reconstruction and fixation techniques, bone−patellar tendon−bone (BPTB) autologous grafts have long been considered the gold standard for the young and functioning patient.^2^ ACL reconstruction with BPTB autograft outcomes have shown superiority in positive quality of bone-to-bone fixation along with excellent clinical and rehabilitation outcomes.^2^ Using BPTB autografts has been reported to have greater tensile strength compared with the native ACL and to have significantly less anterior knee laxity and more stability compared with hamstring autografts.^3^, ^4^, ^5^

BPTB autograft revision rates have been reported to be significantly lower compared with hamstring autografts.^6^ BPTB autografts also have shown improved athletic function and a significant decreased risk in graft rupture at 2 years follow-up.^7^ Aune et al.^8^ reported BPTB autografts to have superior screw fixation compared with hamstring autografts, which allow for more optimal bone-to-bone healing and quicker return to rehabilitation. Many orthopaedic surgeons will opt to not use BPTB autografts because of the required advanced surgical technique and increased risk of anterior knee pain, patella fracture, patella baja, quadriceps shut down, and long-term risk of osteoarthritis.^8^, ^9^, ^10^ Conclusively, BPTB autografts also have been shown to increase the likelihood of graft-tunnel mismatch (GTMM).^11^^,^^12^

GTMM is a condition in which the ACL graft length does not match the intra-articular and tibial tunnel length.^1^ The overall incidence of GTMM during ACL reconstruction has been reported to be 20% when using BPTB autografts and as high as 26% in BPTB allografts, causing significant difficulty for surgeons intraoperatively.^12^^,^^13^ Using BPTB autografts in ACL reconstruction does have a variety of unavoidable risks. These include but are not limited to previous knee joint trauma, degenerative tendon changes, patellar tendinopathy, patella alta, patella baja, Osgood-Schlatter disease, Sinding-Larson-Johansson syndrome, and patients who have obtained preoperative or intraoperative radiographic imaging without proper intra-articular length measurements.^1^ Patients with previous degenerative tendon changes and a graft that is too long are encountered much more commonly, especially when the graft is >50 mm.^13^ When GTMM is encountered with a BPTB autograft, there is an increased risk of bone-to-bone fixation and concern for inadequate bony healing. When the ACL graft is too long, it results in protrusion of the graft out of the tibial bone tunnel and necessitates removal of excess graft, which can result in compromised graft fixation and loss of optimal bone-to-bone healing.^1^ If the graft is too short in GTMM, screw fixation can push the graft up the tibial tunnel, resulting in inadequate tension.^1^

As the result of these increased risks, numerous bailout method options creating longer operative times, greater financial costs, and greater risks to each operative case, many orthopaedic surgeons will opt to use soft-tissue grafts versus BPTB.^1^ It remains imperative that the orthopaedic surgeon know how to address graft tunnel mismatch if performing ACL reconstruction.

We present our technique, in which we use a simple whipstitch for backup fixation that provides reliable tension on the graft without necessitating adjustment of femoral fixation. This will especially be useful to address varying lengths of BPTB autografts, a known phenomenon. Advantages and disadvantages of this technique are described in Table 1.

**Table 1:** Advantages and Disadvantages of Our Technique

Advantages	Disadvantages
Reliable graft tensioning	Additional cost from extra sutures and SwiveLock anchor
Preserves femoral fixation	Requires good bone block quality on both ends
Increased tibial fixation strength	Slightly increased operative time
Flexibility in graft length variability	Potential for suture failure if not properly secured
Minimizes excessive bone removal	Learning curve for surgeons unfamiliar with the technique
Enhanced secondary fixation	Limited long-term data compared with traditional methods
Reduces risk of graft micromotion	Possibility of suture-tunnel conflict if not properly placed
Facilitates faster postoperative rehabilitation	Increased fixation complexity
Prevents tibial tunnel widening	Requires sufficient tibial bone tunnel space
Simple and reproducible technique	May not be suitable for all patients, especially those with poor bone quality
Lower risk of tibial graft slippage	
Maintains bone-to-bone healing benefits	
Can be used with various tunnel lengths	

## Surgical Technique

**Fig 1:**
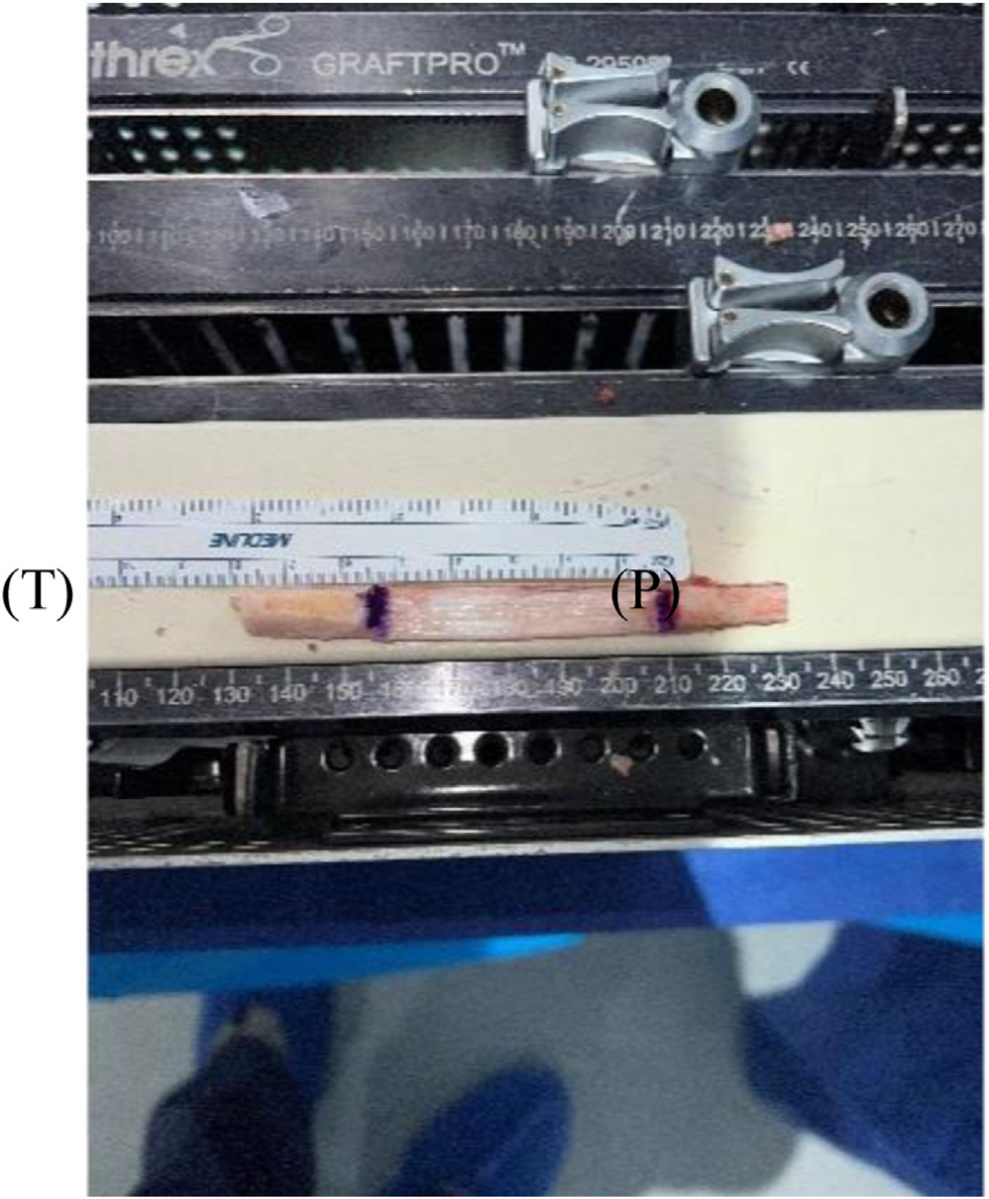
The final harvesting of the bone−patellar tendon−bone autograft has been performed and carefully placed on the back table. The tendinous insertion on the tibial (T) and patellar (P) bone block are marked with an intraoperative marker. The graft is measured with an intraoperative ruler (Medline Industries, Northfield, IL).

**Fig 2:**
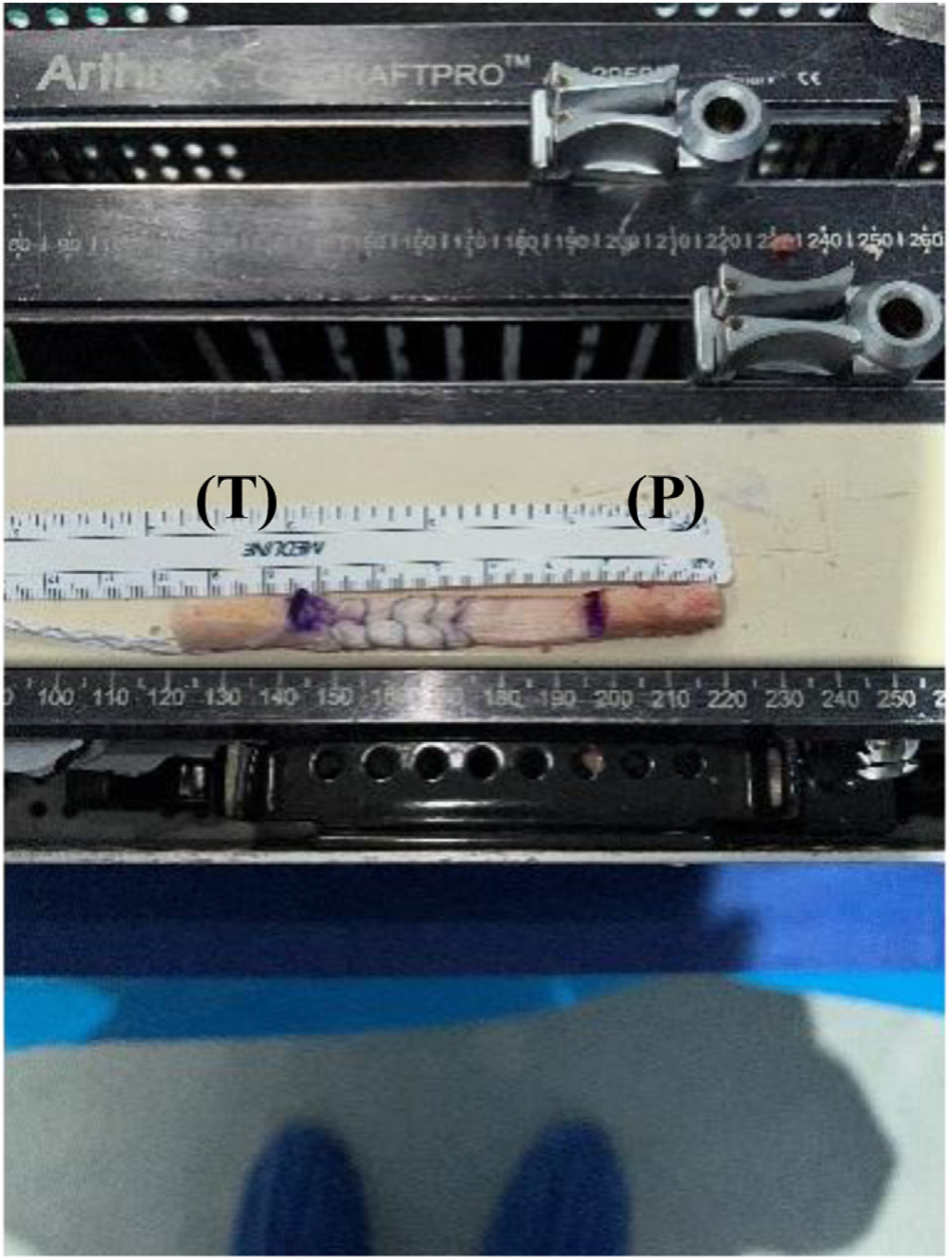
The tibial bone block (T) has now been drilled and standard preparation of the graft through one drill tunnel with a running locking stitch using a 1.3-mm SutureTape (Arthrex) has been performed.

**Fig 3:**
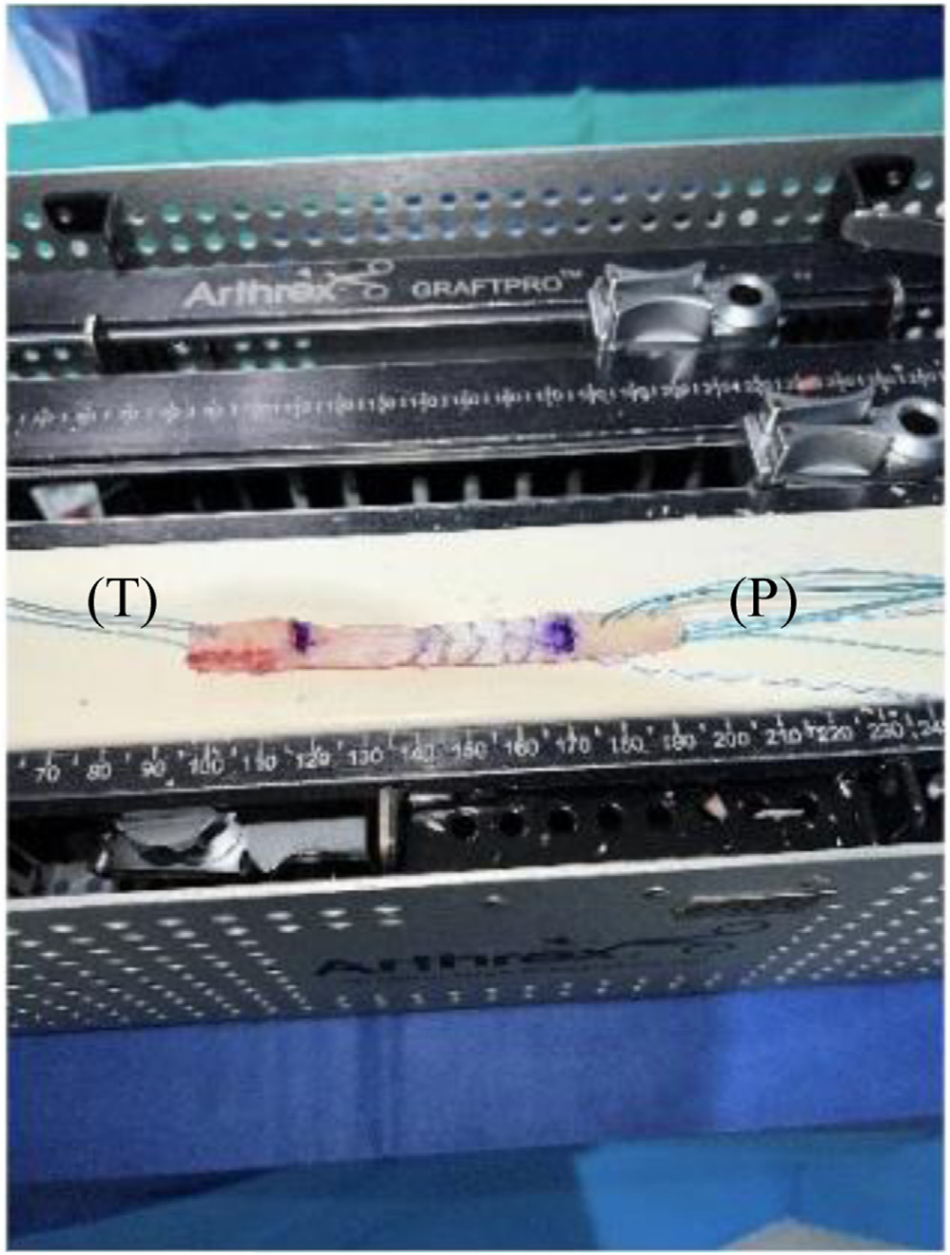
The tibial bone block (T) has now been drilled and standard preparation of the graft through one drill tunnel with a running locking stitch using a 1.3-mm SutureTape (Arthrex) has been performed. A locking stitch has been placed for backup fixation in case of graft tunnel mismatch in the patellar bone block (P) with a 1.3-mm SutureTape fiber loop (Arthrex) 2.0 cm proximal to end of the patellar bone block (P). This is done in a standard running grasping stitch technique. Four to five passes are made with the suture depending on tendon length. The needle is cut leaving 2 free ends of suture.

**Fig 4:**
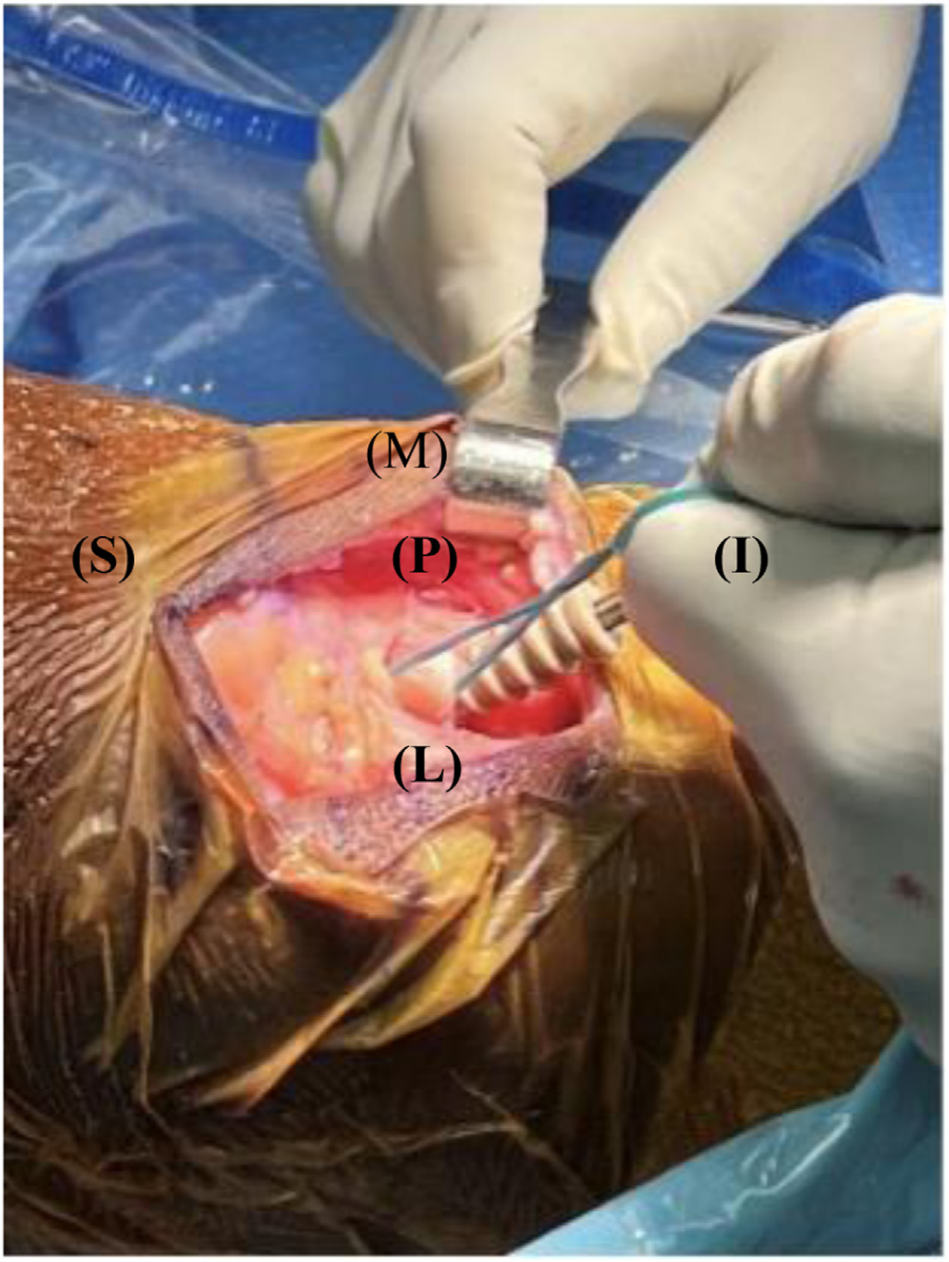
The patient's right knee is depicted with superior (S) to the left, inferior (I) to the right, medial (M) to the top, and lateral (L) to the bottom of the figure. The knee is held in full extension with a 2-finger posterior drawer pressure placed on the tibia and the previously placed locking suture on the patellar bone block (P) is fully tensioned. A 9 × 20-mm Fast Thread Peek Interference Screw (Arthrex) is placed and terminally tightened. There is excess patellar bone block (P) that can now be removed as a result of the graft tunnel mismatch.

**Fig 5:**
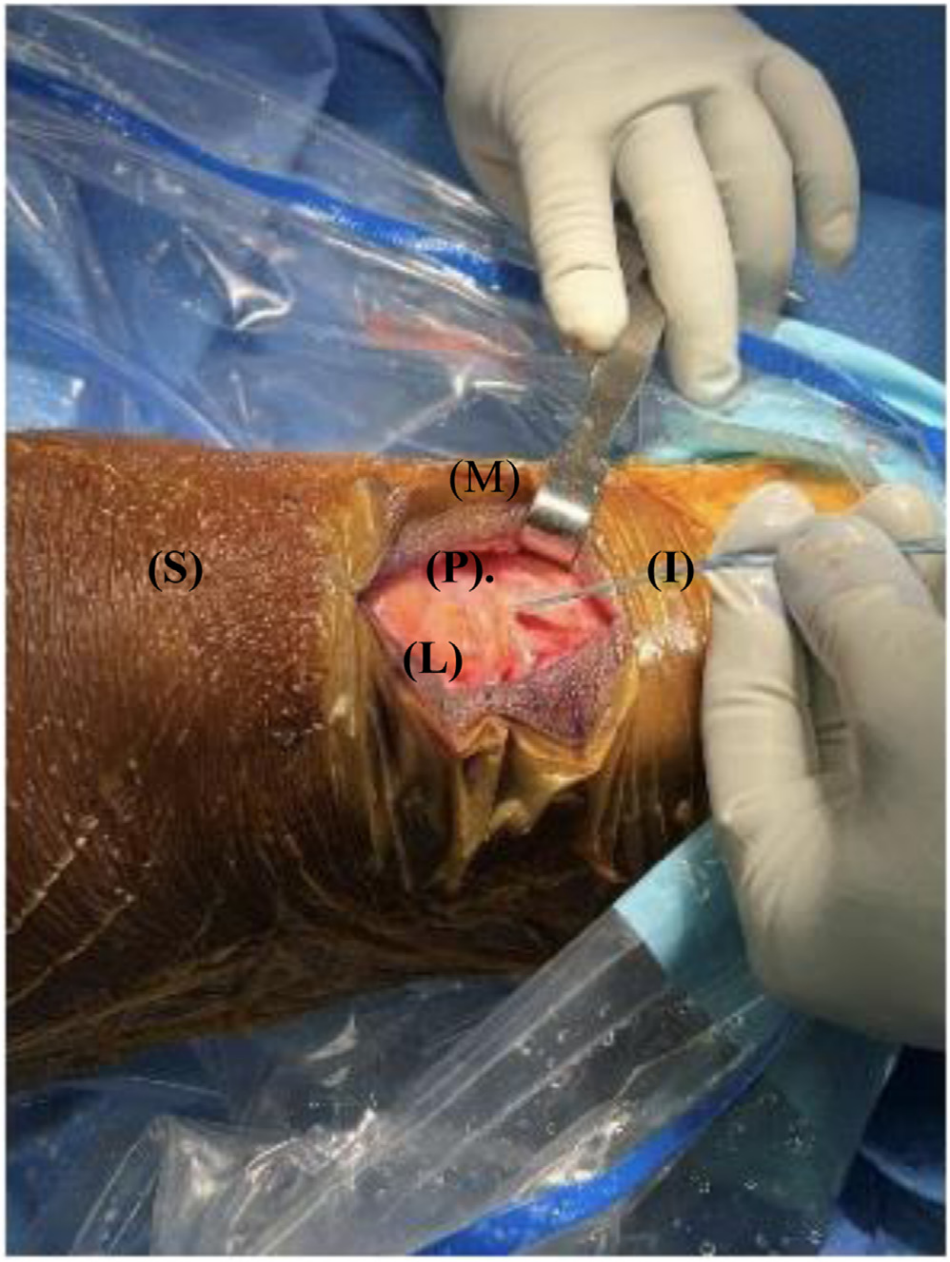
The patient's right knee is depicted with superior (S) to the left, inferior (I) to the right, medial (M) to the top, and lateral (L) to the bottom of the figure. The 9 × 20-mm Fast Thread Peek Interference Screw (Arthrex) in the tibial tunnel is represented next to the patellar bone block (P). The remaining sutures from the patellar bone block (P) are represented in the figure in preparation to be placed into a 4.75-mm SwiveLock (Arthrex).

**Fig 6:**
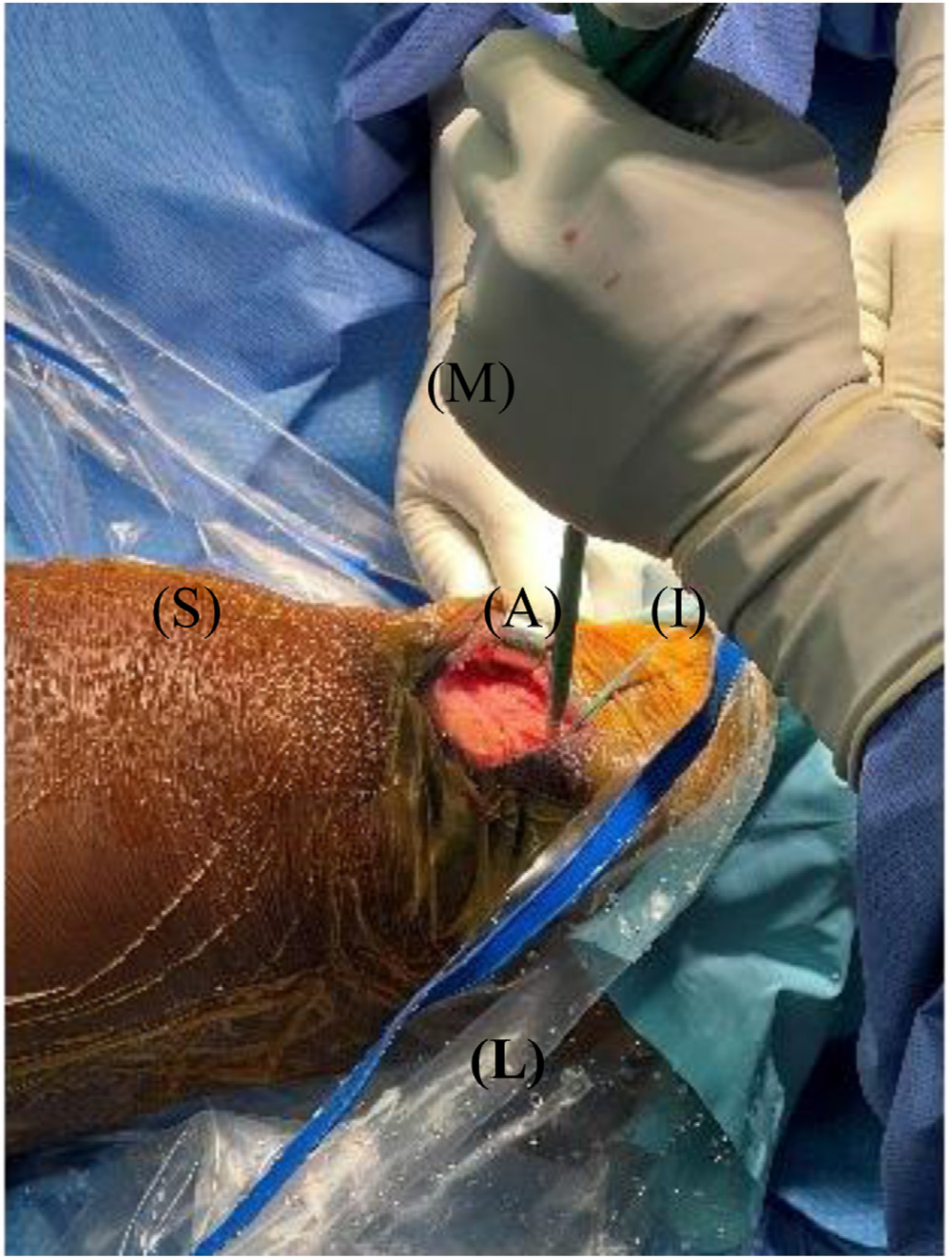
The patient's right knee is depicted with superior (S) to the left, inferior (I) to the right, medial (M) to the top, and lateral (L) to the bottom of the figure. The remaining sutures from the patellar bone block (P) are placed into a 4.75-mm SwiveLock (Arthrex) and the SwiveLock (A) is placed into the tibia after drilling and tapping with the knee in full extension.

Our surgical technique is shown in Video 1. Standard harvesting of BPTB graft autograft is first performed (Fig 1). Standard preparation of the graft is then next carried out. We prefer one suture passed through a drill hole in tibial bone block (Fig 2) and 3 sutures passed through separate holes in patellar bone block. The tendinous portion is then measured, and the graft is marked with an operative marking pen. If this length is less than 50 mm, the decision is made to place a locking suture for backup (Fig 3). This is done using a 1.3-mm SutureTape fiber loop (Arthrex, Naples, FL) 2 cm proximal to patellar bone block. This is done in a standard running grasping stitch technique. Four to five passes are made with the suture, depending on tendon length. The needle is cut, leaving 2 free ends of suture. Standard ACL reconstruction is then carried out, first securing tibial bone block in femur (we prefer 9 × 20-mm Fast Thread Peek Interference Screw; Arthrex). Examination of patellar bone block and amount of protruding bone block are next examined on the tibial side. If this amount is <5 mm, standard fixation is used with a 9 × 30-mm screw with no backup fixation (Fig 4). However, if >5 mm of bone is protruding, then an interference screw is first placed in the tibia. A sagittal saw is used to trim excess bone off the protruding patellar bone block, with care taken to protect sutures. Remaining sutures from bone block in addition to the 2 ends of SutureTape are then placed in a 4.75-mm SwiveLock (Arthrex) at desired degree of knee flexion; we prefer full extension (Figs 5 and 6). Knee wounds are then closed in standard fashion.

## Discussion

ACL reconstruction remains a technically demanding procedure, and optimizing fixation techniques is critical to achieving successful outcomes. BPTB autografts have long been considered the gold standard for ACL reconstruction, particularly in young, high-demand patients as a result of their superior bone-to-bone healing and biomechanical properties However, one significant intraoperative challenge associated with BPTB autografts is GTMM, which occurs when the graft length does not correspond with the intra-articular and tibial tunnel dimensions, leading to suboptimal fixation.

In a recent study, Dwyer et al.^14^ investigated the incidence of GTMM in ACL reconstruction using anteromedial portal drilling and found a 6% mismatch rate. Their study emphasized that tibial tunnel distances exceeding the patellar tendon length by more than 5 mm increased the risk of GTMM to 9%, whereas those within a 5-mm range had only a 2% incidence of GTMM. Traditional management strategies for GTMM include femoral tunnel recession, free bone block techniques, and altering tunnel drilling angles, but these approaches can increase surgical time and introduce additional complexities.^14^ Our technique provides an effective alternative by using a simple whipstitch for backup fixation, which ensures reliable tension on the graft without necessitating femoral fixation adjustments.

Furthermore, in a comprehensive review, Battaglia^15^ discusses various intraoperative challenges in ACL reconstruction, highlighting GTMM as a key complication affecting graft placement and fixation stability. The author outlines different management strategies, including tunnel length modifications and bone block adjustments, but noted that additional fixation techniques could be beneficial in addressing GTMM.^15^ Our proposed whipstitch backup fixation aligns with these recommendations, because it provides an effective method to manage GTMM without requiring complex tunnel modifications. This technique complements existing methods while preserving bone-to-bone healing and biomechanical integrity.

In addition, Eskew et al.^1^ conducted a study on GTMM in BPTB ACL reconstruction, reporting an overall GTMM incidence of 20%, which was even greater in cases using BPTB allografts (up to 26%). Their findings emphasized the importance of preoperative planning, intraoperative flexibility, and innovative fixation techniques to mitigate GTMM-related complications. The study also highlighted risk factors such as patella alta, patella baja, and improper tunnel placement, reinforcing the need for adaptable solutions like our whipstitch backup fixation technique.^1^ Our approach directly addresses these concerns by offering an intraoperative solution that ensures adequate graft tension and stability without requiring tunnel modifications.

Moreover, a recent study published in *The Knee Journal* (2020) assessed the prevalence of GTMM in ACL reconstruction among leading orthopaedic surgeons, identifying the frequency with which GTMM is encountered and the diverse techniques used to address it.^16^ The study reaffirmed that GTMM remains a common challenge in ACL surgery, highlighting the need for continued innovation in surgical techniques.^16^ Our whipstitch backup fixation technique directly contributes to this ongoing evolution by providing a reliable, reproducible method for addressing GTMM intraoperatively while preserving the biomechanical advantages of BPTB autografts.

Compared with existing GTMM management techniques, our approach offers several advantages. First, it preserves bone-to-bone healing, maintaining the biomechanical integrity of BPTB autografts. Second, it minimizes excessive bone removal, which can compromise graft fixation and healing. In addition, by providing a secondary fixation mechanism, our technique reduces the risk of tibial tunnel widening and graft micromotion, potentially improving long-term graft stability.

One notable benefit of our technique is its ease of reproducibility. Unlike complex bailout procedures such as free bone block techniques, our method requires only a simple whipstitch using a 1.3-mm SutureTape fiber loop, making it an accessible option for orthopaedic surgeons of varying experience levels. Moreover, our approach allows surgeons to address varying BPTB graft lengths intraoperatively, avoiding the need for preoperative adjustments on the basis of estimated patellar tendon lengths.

However, our technique is not without limitations. As with any additional fixation step, there is a small increase in operative time and material cost because of the requirement for additional suture anchors. In addition, long-term clinical outcomes comparing our method with other GTMM management techniques remain to be studied, and future research should evaluate its biomechanical stability in cadaveric and clinical settings. Further studies with larger sample sizes and long-term follow-up are warranted to assess the efficacy of this technique in reducing GTMM-related complications.

In conclusion, GTMM remains a significant challenge in BPTB ACL reconstruction, particularly when tunnel lengths do not match graft dimensions. This method is simple, effective, and preserves the advantages of BPTB autografts while minimizing the need for tunnel adjustments. As ACL reconstruction techniques continue to evolve, incorporating reliable backup fixation strategies will be crucial in optimizing surgical outcomes and improving patient recovery.

## Disclosures

All authors (N.M., G.M., J.R.E.) declare that they have no known competing financial interests or personal relationships that could have appeared to influence the work reported in this paper.

## Supplementary Data

Video 1 Standard preparation of the bone patellar tendon bone autograft is carried out. One suture passed through a drill hole in tibial bone block and 3 sutures passed through separate holes in patellar bone block. The tendinous portion is then measured, and the graft is marked with an operative marking pen. If this length is less than 50 mm, the decision is made to place a locking suture for backup. This is done using a 1.3-mm SutureTape fiber loop (Arthrex, Naples, FL) 2 cm proximal to patellar bone block. This is done in a standard running grasping stitch technique. Four to five passes are made with the suture depending on tendon length. The needle is cut leaving 2 free ends of suture. Standard ACL reconstruction is then carried out, first securing tibial bone block in femur (we prefer 9 ×20-mm Fast Thread Peek Interference Screw (Arthrex). Examination of patellar bone block and amount of protruding bone block are next examined on the tibial side. If this amount is <5 mm, standard fixation is used with a 9 × 30-mm screw with no back up fixation. However, if >5 mm of bone is protruding then an interference screw is first placed in tibia. A sagittal saw is used to trim excess bone off protruding patellar bone block taking care to protect sutures. Remaining sutures from bone block in addition to the 2 ends of SutereTape are then placed in a 4.75-mm SwiveLock (Arthrex) at desired degree of knee flexion, we prefer full extension.
